# The regulatory mechanism of cyclic GMP-AMP synthase on inflammatory senescence of nucleus pulposus cell

**DOI:** 10.1186/s13018-024-04919-1

**Published:** 2024-07-22

**Authors:** Rui Sun, Feng Wang, Cong Zhong, Hang Shi, Xin Peng, Jia-Wei Gao, Xiao-Tao Wu

**Affiliations:** 1 Department of Orthopedics, School of Medicine, Zhongda Hospital, Southeast University, NO. 87 Ding Jia Qiao, Nanjing, Jiangsu Province 210003 China; 2 School of Medicine, Southeast University, Nanjing, Jiangsu Province 210003 China

**Keywords:** cGAS, Intervertebral disc degeneration, Nucleus pulposus cells, Senescence, IL-1β, NF-κB pathway

## Abstract

**Background:**

Cellular senescence features irreversible growth arrest and secretion of multiple proinflammatory cytokines. Cyclic GMP-AMP synthase (cGAS) detects DNA damage and activates the DNA-sensing pathway, resulting in the upregulation of inflammatory genes and induction of cellular senescence. This study aimed to investigate the effect of cGAS in regulating senescence of nucleus pulposus (NP) cells under inflammatory microenvironment.

**Methods:**

The expression of cGAS was evaluated by immunohistochemical staining in rat intervertebral disc (IVD) degeneration model induced by annulus stabbing. NP cells were harvested from rat lumbar IVD and cultured with 10ng/ml IL-1β for 48 h to induce premature senescence. cGAS was silenced by cGAS specific siRNA in NP cells and cultured with IL-1β. Cellular senescence was evaluated by senescence-associated beta-galactosidase (SA-β-gal) staining and flow cytometry. The expression of senescence-associated secretory phenotype including IL-6, IL-8, and TNF-a was evaluated by ELISA and western blotting.

**Results:**

cGAS was detected in rat NP cells in cytoplasm and the expression was significantly increased in degenerated IVD. Culturing in 10ng/ml IL-1β for 48 h induced cellular senescence in NP cells with attenuation of G1-S phase transition. In senescent NP cells the expression of cGAS, p53, p16, NF-kB, IL-6, IL-8, TNF-α was significantly increased while aggrecan and collagen type II was reduced than in normal NP cells. In NP cells with silenced cGAS, the expression of p53, p16, NF-kB, IL-6, IL-8, and TNF-α was reduced in inflammatory culturing with IL-1β.

**Conclusion:**

cGAS was increased by NP cells in degenerated IVD promoting cellular senescence and senescent inflammatory phenotypes. Targeting cGAS may alleviate IVD degeneration by reducing NP cell senescence.

## Introduction

Low back pain is the primary cause of productivity loss worldwide and is ranked as the leading disability-causing condition globally. The incidence of low back pain has seen a 50% increase from 1990 to 2019 [1–3]. Intervertebral disc (IVD) degeneration is a common health problem and one of the main causes of low back pain, characterized by loss of cell viability, destruction of water-bonding extracellular matrix, and elevation of inflammatory cytokines [4–7]. Cellular senescence is one of the mechanisms that reduce cell viability and demonstrate proinflammatory phenotype [8]. In senescent cells the cell cycle irreversibly stays in the G1-S transition phase and cells no longer divide and proliferate [9]. Additionally, senescent cells exhibit upregulated expression and secretion of multiple cytokines. This leads to the development of an inflammatory senescence-associated secretory phenotype (SASP) and creates a degenerating environment for neighboring normal cells [10–12]. SASP is characterized by the upregulation of inflammatory cytokines such as IL-6, IL-8, and TNF-α initiating a cascade of inflammatory responses [13, 14]. Accumulating evidence shows that the proportion of senescent disc cells is increased in degenerated IVD, and targeting cellular senescence alleviates IVD degeneration [6, 15–17].

Cyclic GMP-AMP synthase (cGAS) is one of the key regulators in demonstrating the inflammatory SASP [18, 19]. In senescent cells with DNA damage and cytoplasmic chromatin fragments, cGAS acts as intracellular DNA sensor to induce the production of type I interferon (IFN I). IFN I activates the stimulator of interferon genes (STING) to produce the second message cyclic GMP -AMP (cGAMP), which activates NF-κB pathway to express SASP [12, 15–17, 19]. By detecting DNA damage through the DNA-sensing pathway, cGAS initiates the expression of inflammatory genes, leading to cellular senescence [20–22]. It has been increasingly recognized that cGAS-STING signal is essential for the inflammatory SASP in cellular senescence induced by various pro-senescent pathogenesis such as oxidative stress, radiation, oncogene activation, and chronic inflammation [22–25].

In degenerated IVD, there is an accumulation of senescent cells, which contributes to an upregulation in the expression of pro-inflammatory cytokines such as IL-1, IL-6, and TNF-α [15–17, 26, 27]. However, to date, it remains unknown whether the cGAS-STING pathway is also essential for inflammatory SASP in NP cells. In this study, cGAS was silenced in rat NP cells to investigate the change of SASP under inflammation stimuli. By identifying the key regulators of NP cell senescence and SASP, our study may help to provide new target for anti-senescence therapy aimed at regenerating IVD.

## Materials and methods

### IVD degeneration model in rat

Animal studies were approved by Laboratory Animal Care and Use Committee of Southeast University (20,210,221,019). 16 Male Sprague-Dawley rats of 8-weeks old (250 g) were provided by Southeast University Laboratory Animal Centre. For inducing IVD degeneration, 8 rats were anesthetized with pentobarbital and placed in prone position with the tail skin sterilized by ethanol. The 5–6, 6–7, 7–8 discs of coccygeal were identified and IVD degeneration was induced by annulus puncture in the 6–7 disc. A 21G needle was punctured vertically to a depth of 5 mm into disc and rotated 360° for 30s. The 5–6 and 7–8 discs remained intact and were used as control. 8 weeks post puncture the IVD degeneration was evaluated by MRI with BioSpec 7T/20 USR spect (Bruker BioSpin, Billerica, MA, USA; T2 TurboRARE, TE 25 ms, TR 2000 ms, thickness 0.7 mm). After MRI the 5–6, 6–7, and 7–8 vertebrae-disc-vertebrae unites were collected and fixed in 4% paraformaldehyde, followed by dehydration with graded ethanol series and embedment in paraffin wax to prepare coronal sections of 3 μm thick for histological and immunohistochemical staining.

### Histological staining and immunohistochemical detection of cGAS in degenerated IVD

For histological evaluation of IVD degeneration, the sections of vertebrae-disc-vertebrae unites were rehydrated with graded ethanol for Hematoxylin-Eosin (HE) staining and Safranin-O (SO) staining. Histological analysis was performed using a microscope and evaluated based on a histological grading system [28]. The grading system assigned a histologic score to each disc sample, with a score of 5 indicating a normal disc, a score of 6–15 indicating degeneration. For immunohistochemical staining of cGAS, the rehydrated sections were heated to 90 °C for 20 min in sodium citrate buffer (10 mM sodium citrate, PH to 6.0, 0.05% Tween 20) for antigen retrieval. The endogenous peroxidase was eliminated by reaction with 3% hydrogen peroxide in methanol. After blocking with 5% bovine serum albumin in Tris-buffered saline, the sections were incubated with rabbit anti-cGAS (1:100; Arigobio) primary antibody at 4 °C overnight and incubated with Goat Anti-Rabbit IgG H&L (Abcam, USA) at room temperature for 1 h. The binding of anti-cGAS antibody was visualized by ImmPACT DAB substrate (SK-4105; Vector Labs, Burlingame, CA, USA). Cell nuclei were counterstained with hematoxylin. Expression of cGAS was quantified by counting the positive staining cells under light microscopy.

### NP cells culturing and induction of cellular senescence

NP cells were isolated from 8 male Sprague-Dawley rats. After euthanized with pentobarbital, the lumber spine was collected aseptically and IVD were exposed to collect the inner gel-like NP tissue. Low glucose Dulbecco’s modified Eagle’s medium (DMEM/F-12; Gibco, China) containing 0.1% collagenase type II (SigmaeAldrich, USA) and 1% fetal bovine serum (FBS; Gibco, Shanghai, China) was used to digest NP for 4–6 h at 37 ℃. The partially digested NP were maintained in DMEM/F-12 containing 10% FBS and antibiotics, cultured under 95% humidity, 21% O_2_, 5% CO_2_ at 37 ℃. Confluent cells were lifted using 0.25% Trypsine EDTA (1 mM) solution (Life Technologies, China) and sub-cultured (1:2) to collect passage 1 (P1), P2, and P3 NP cells. The P3 NP cells were utilized in the following experiments unless otherwise indicated.

Cellular senescence was induced by culturing NP cells with different concentration of IL-1β (0, 5, 10, 20 ng/ml) for 24, 48, and 72 h. After culturing with IL-1β, cell proliferation was evaluated by cell counting kit-8 (CCK-8, Beyotime, China). Briefly NP cells were plated in 96-well plates at a density of 1500 cells per well for 24 h and cultured with IL-1β solution. CCK-8 reagent at a concentration of 10% was added to each well, cultured in 5% CO_2_ at 37℃ for 4 h. The absorbance was measured at a wave length of 450 nm with Multiskan MK3 (Thermo Scientific, USA) to quantify proliferation viability. Cellular senescence was evaluated by senescence-associated beta-galactosidase (SA-β-gal) staining. Briefly, NP cells were planted in 6-well plates and cultured with 10 ng/ml IL-1β solution for 48 h. After fixed in 4% paraformaldehyde and washed with PBS, NP cells were incubated in SA-β-gal working solution at 37℃ for 12 h. Cells were visualized using the inverted phase contrast microscope (Olympus CKX41, Japan), images were captured using digital camera and Q-Capture pro.6.0 system (Media Cybernetics and Qimaging Corporation, USA), and the percentage of SA-β-gal positive cells was calculated by counting 100 cells.

### Silencing cGAS in NP cells

NP cells were plated in 6-well plates at 5 × 10^5^ cells per well and transfected with cGAS-targeting small interfering RNAs (siRNAs) according to the manufacturer’s protocol (Genechem, Shanghai, China). After six hours of transfection, NP cells were serum-starved for 24 h and treated with 10 ng/ml IL-1β solution for 48 h. The efficiency of cGAS transfection was evaluated by immunofluorescence staining. After culturing in IL-1β solution the NP cells were fixed in 4% paraformaldehyde and permeabilized with 0.2% Triton X-100 in PBS. After blocking with 5% goat serum albumin in TBST (50 mM Tris, pH 7.6, 150 mM NaCl, 0.1% Tween 20), NP cells were incubated with primary antibodies against cGAS (1:100; Arigobio) at 4 °C overnight. After washing the NP cells were reacted with Alexa Fluor 647 goat anti-rabbit IgG (1:1,000; Abcam) secondary antibodies and labeled with DAPI. Negative control was performed following the same procedure without incubation of primary antibodies. The stained cells were imaged using fluorescence microscope (EVOS FL Imaging System, Life Technologies, US). To evaluate the impact of cGAS gene knockdown on the degree of senescence in NP cells, the senescent rate of cGAS-silenced NP cells was evaluated by SA-β-gal staining and cell cycle distribution analysis. Briefly, after treated with IL-1β, NP cells were collected and suspended in 500 ml propidium iodide (PI) working solution of Cell Cycle Detection Kit (KeyGen, China). The percentage of cells in G1 and S-phase were measured by flow cytometric analysis. FACScan cell sorter (Becton Dickinson, Mountain View, USA), ModFit LT software (Verity Software House, Topsham, USA) and GraphPad Prism 7 Software (GraphPad Software, San Diego, CA, USA) were used to quantify cell cycle distribution.

### Anti-senescence effect of silencing cGAS in NP cells

To study the anti-senescence effect of cGAS in NP cells, both normal and cGAS-silenced NP cells were treated with 10 ng/ml IL-1β solution for 48 h. The expression of senescence signals and phenotype molecules was evaluated by western blotting. Briefly, after culturing with IL-1β, the total protein of NP cells was isolated using a whole protein extraction kit (Keygen Biotech, Nanjing, China) and the protein concentration was measured by the BCA protein assay kit (Beyotime). Proteins were resolved on 10% SDS-PAGE gels and transferred by electroblotting onto PVDF membranes (EMD Millipore Corporation, US). The membranes were blocked with 5% nonfat dry milk in TBST and incubated at 4 °C overnight with primary antibodies: cGAS (1:500; Abcam, Cambridge, UK), p53 (1:1,000; Cell Signaling), p16 (1:1,000; Abcam), NF-κB (1:1,000; Cell Signaling), phosphorylated (p-)NF-κB (1:2,000; Abcam), Aggrecan (1:1,000; Affinity Biosciences, USA), Collagen II (1:2,000; Proteintech, China), IL-6 (1:1000; R&D Systems, USA), IL-8 (1:1,000; Proteintech), TNF-α (1:1,000; Proteintech), GAPDH(1:1000; Abcam). Following incubation with peroxidaseconjugated secondary antibody (A0208, Beyotime, China), the immunolabeling was detected using the Super Signal West Pico Chemiluminescent Substrate (Thermo Scientific, US). The expression level of senescence signals and phenotype molecules was quantified by densitometric analysis and normalized to the level of GAPDH. The secretion of IL-6, IL-8, TNF-α in the culturing medium was evaluated by ELISA kits (Servicebio). After culturing with IL-1β, the culture medium of NP cells was centrifuged to collect the supernatant. The collected medium was diluted at 1:2 and added into enzyme plate to measure the OD value of each well at 450 nm. The concentration of IL-6, IL-8, TNF-α was quantified based on the standard curves of targeted protein.

### Statistical analysis

SPSS v19.0 software (IBM, Armonk, New York, USA) was used to perform statistical analysis. All quantitative data were obtained from three independent experiments and presented as mean ± standard deviation. Differences between two groups were analyzed by unpaired *t* test. Differences among multiple groups were analyzed by one-way ANOVA followed by Tukey’s multiple comparisons test. Statistical significance was defined as P value < 0.05.

## Result

### cGAS was increased in NP cells of degenerated rat IVD

**Fig. 1:**
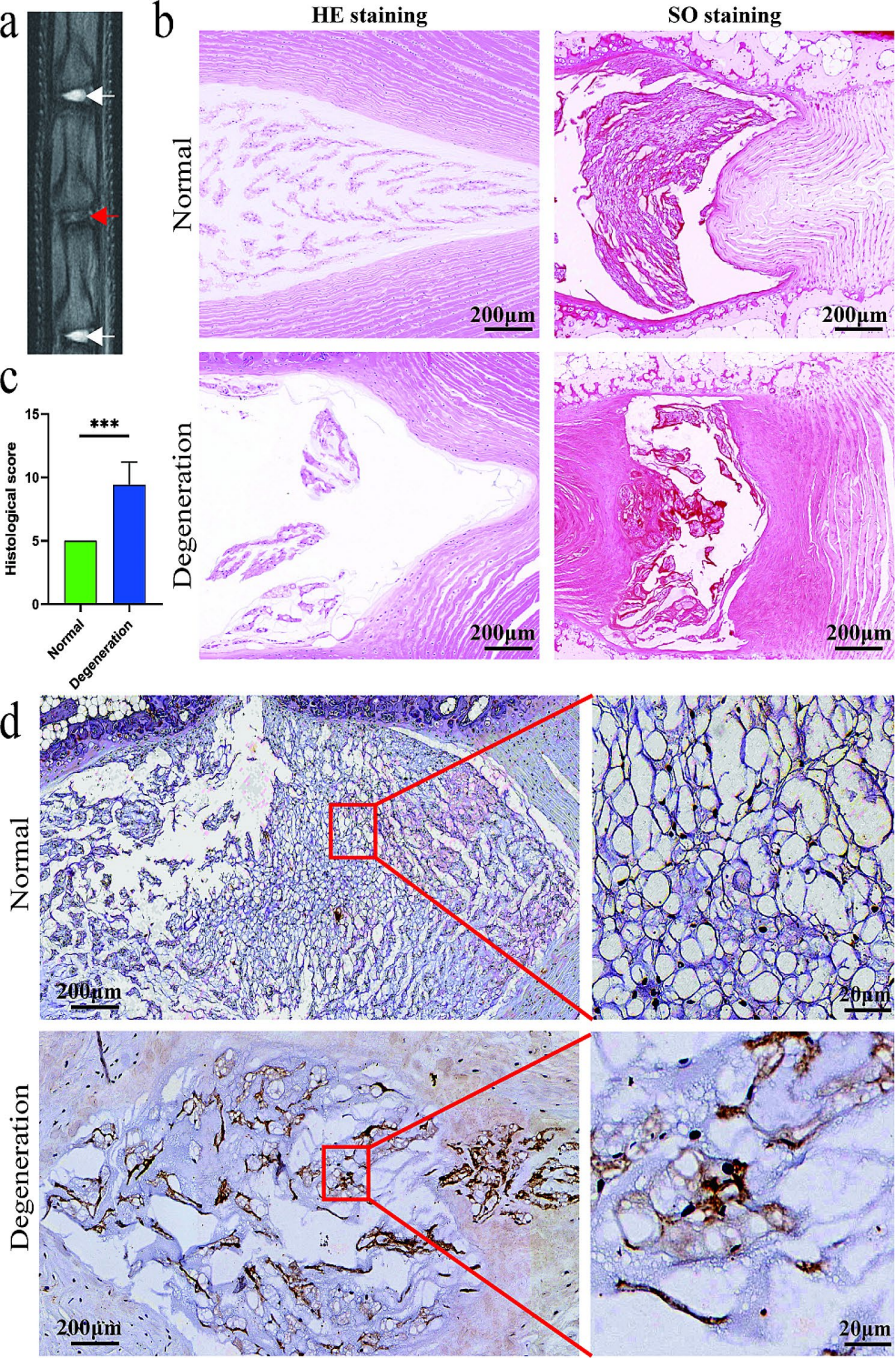
cGAS expression was increased in NP cells of degenerated rat IVD. (**a**) MR imaging of rat coccygeal 5–6, 6–7 and 7–8 IVD 8 weeks post annulus puncture. The punctured coccygeal 6–7 disc (red arrow) showed significant loss of T2-weighted signal than the intact coccygeal 5–6 and 7–8 discs (white arrow). (**b**, **c**) HE staining and SO staining of normal and degenerated IVD induced by annulus puncture. The punctured disc showed histological degeneration with reduced NP tissue content and distorted boundary between NP and annulus fibrosis. Scare bar: 200 μm. (**d**) Immunohistochemical staining of cGAS expression in normal and degenerated IVD induced by annulus puncture. The expression of cGAS was stained brown mainly in the cytoplasm of NP cells. In degenerated IVD the cGAS expression was significantly increased than in normal IVD. Scare bar: 200 μm

8 weeks after annulus puncture, the punctured rat disc showed significant loss of T2-weighted signal in NP on MRI (Fig. 1a). On HE staining and SO staining, the punctured disc showed degeneration with reduced NP tissue content and distorted boundary between NP and annulus fibrosis (Fig. 1b). On immunohistochemical staining, cGAS was seldom detected in NP of normal rat IVD. The expression of cGAS was significantly increased in the cytoplasm of NP cells in the punctured and degenerated IVD (Fig. 1d).

### IL-1β induced cellular senescence and expression of cGAS in NP cells

**Fig. 2:**
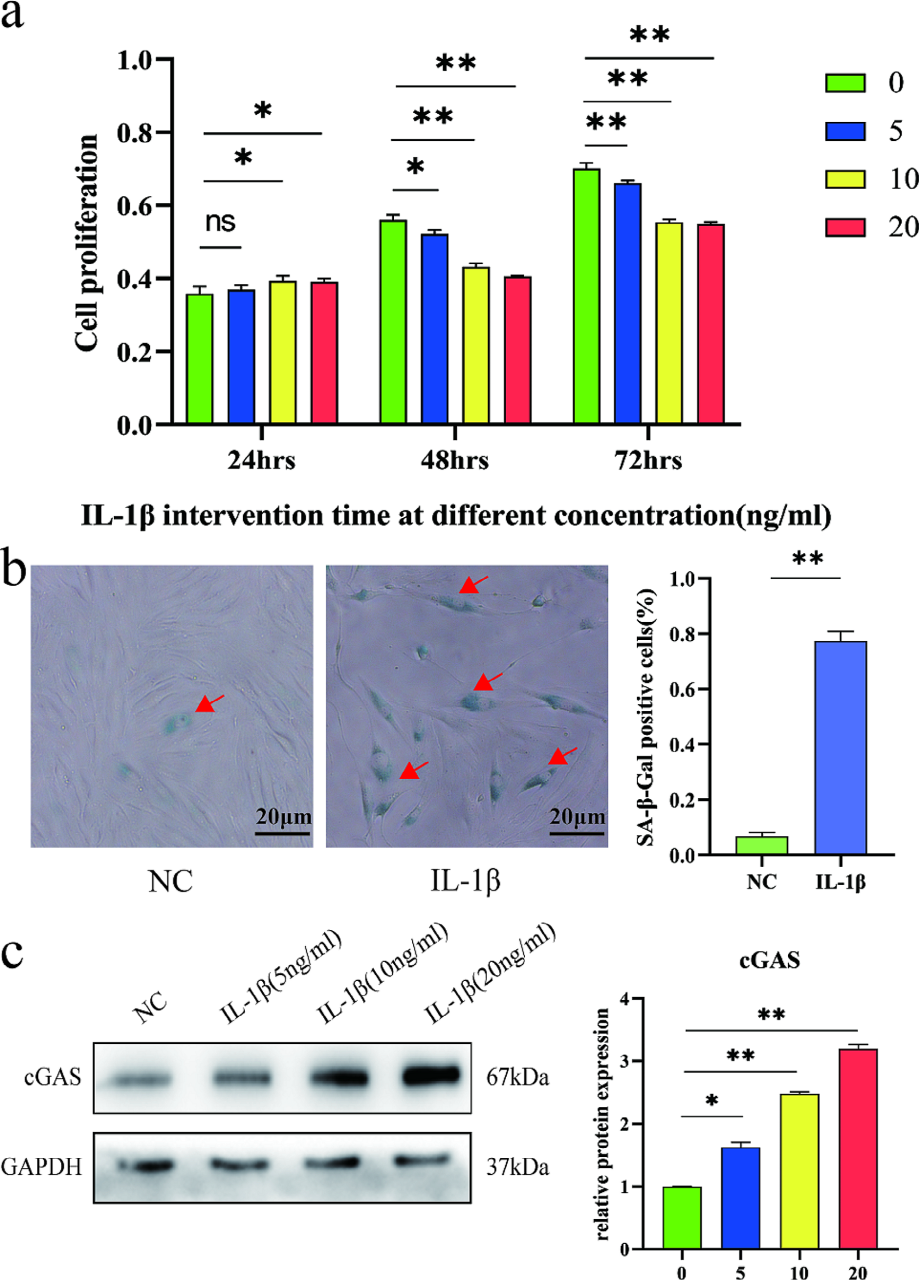
IL-1β induced cellular senescence and expression of cGAS in NP cells. (**a**) CCK-8 analysis of NP cells under 0–20 ng/ml IL-1β for 24–72 h. The cell proliferation activity of NP cells showed time- and dose-dependent reduction in culture with IL-1β. (**b**) SA-β-gal staining of NP cell senescence induced by 10 ng/ml IL-1β treatment for 48 h. The senescent NP cells were stained light green (red arrow). Scare bar: 20 μm. Culturing with 10 ng/ml IL-1β for 48 h induced significantly higher senescent rate than normal NP cells. (**c**) Western blot analysis of cGAS expression in NP cells exposed to 0–20 ng/ml IL-1β for 48 h. Quantification of immunoblots showed a dose-dependent increased expression of cGAS in culture with IL-1β. *p<0.05, **p<0.01, ns, not statistically significant

Cultured with IL-1β, NP cells showed time- and dose-dependent reduction of cell proliferation activity by CCK-8 analysis (Fig. 2a). At both 48 h and 72 h of intervention, the decrease in cell proliferation activity was significantly consistent for IL-1β concentrations of 10 ng/ml and 20 ng/ml compared to the control group. To avoid excessive stimulation, a lower concentration of 10 ng/ml was chosen for the intervention. Under the intervention concentration of 10 ng/ml, the decrease in cell proliferation activity at 48 h and 72 h was basically consistent with the control group. To increase experimental efficiency, a shorter duration of 48 h was chosen as the intervention time. As compared to normal NP cells, the NP cells exposed to 10 ng/ml IL-1β for 48 h showed significantly higher positive SA-β-gal staining (Fig. 2b). When cultured with 5, 10, 20 ng/ml IL-1β for 48 h, NP cells showed dose-dependent increased expression of cGAS (Fig. 2c).

### Silencing cGAS alleviated IL-1β induced NP cell senescence

**Fig. 3:**
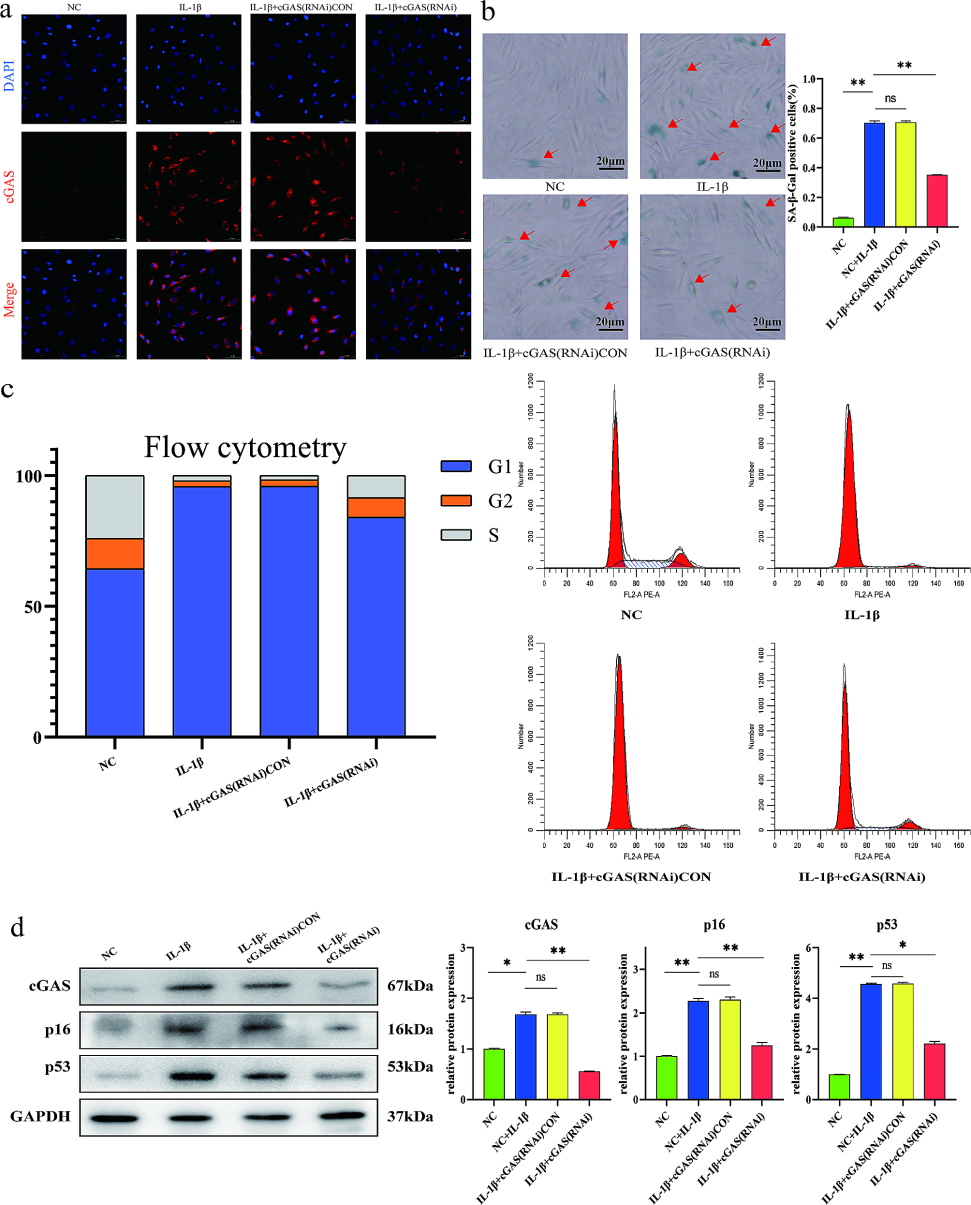
Silencing cGAS alleviated IL-1β induced NP cell senescence. (**a**) Immunofluorescent staining of cGAS expression in NP cells. cGAS expression was increased by culturing with 10 ng/ml IL-1β for 48 h. As compared to non-specific control siRNAs, cGAS-targeting siRNAs significantly silenced the IL-1β-induced cGAS expression in NP cells. Scare bar: 50 μm. (**b**) SA-β-gal staining of NP cells cultured with 10 ng/ml IL-1β for 48 h. The NP cells transfected with cGAS-targeting siRNAs showed significantly lower senescent rate than with non-specific control siRNAs. The senescent NP cells were stained light green (red arrow). Scare bar: 20 μm. (**c**) Cell cycle analysis by flow cytometry in NP cells. Culturing with 10 ng/ml IL-1β for 48 h induced G1-S phase attenuation in normal or control siRNAs transfected NP cells, which was alleviated by cGAS-targeting siRNAs. (**d**) Western blot analysis of cGAS, p16 and p53 expression in NP cells treated with 10ng/ml IL-1β for 48 h. IL-1β induced cGAS, p16 and p53 expression in NP cells, which was significantly reduced by silencing cGAS with cGAS-targeting siRNAs. *p<0.05, **p<0.01, ns, not statistically significant

The cGAS gene expression was silenced by cGAS-targeting siRNAs in NP cells. As compared to normal NP cells and the NP cells transfected with non-specific control siRNAs, the expression of cGAS was significantly reduced when cultured with 10 ng/ml IL-1β for 48 h (Fig. 3a). On SA-β-gal staining, the IL-1β induced senescence was lower in the NP cells with silenced cGAS expression (Fig. 3b). On flow cytometry, IL-1β induced attenuation of G1-S phase transition in NP cells (Fig. 3c). As compared to the NP cells transfected with non-specific control siRNAs (si-con), the G1-S phase attenuation and expression of p53 and p16 were significantly reduced in the NP cells with silenced cGAS (Fig. 3d).

### Silencing cGAS reduced the expression of inflammatory secretory phenotype in NP cells

**Fig. 4:**
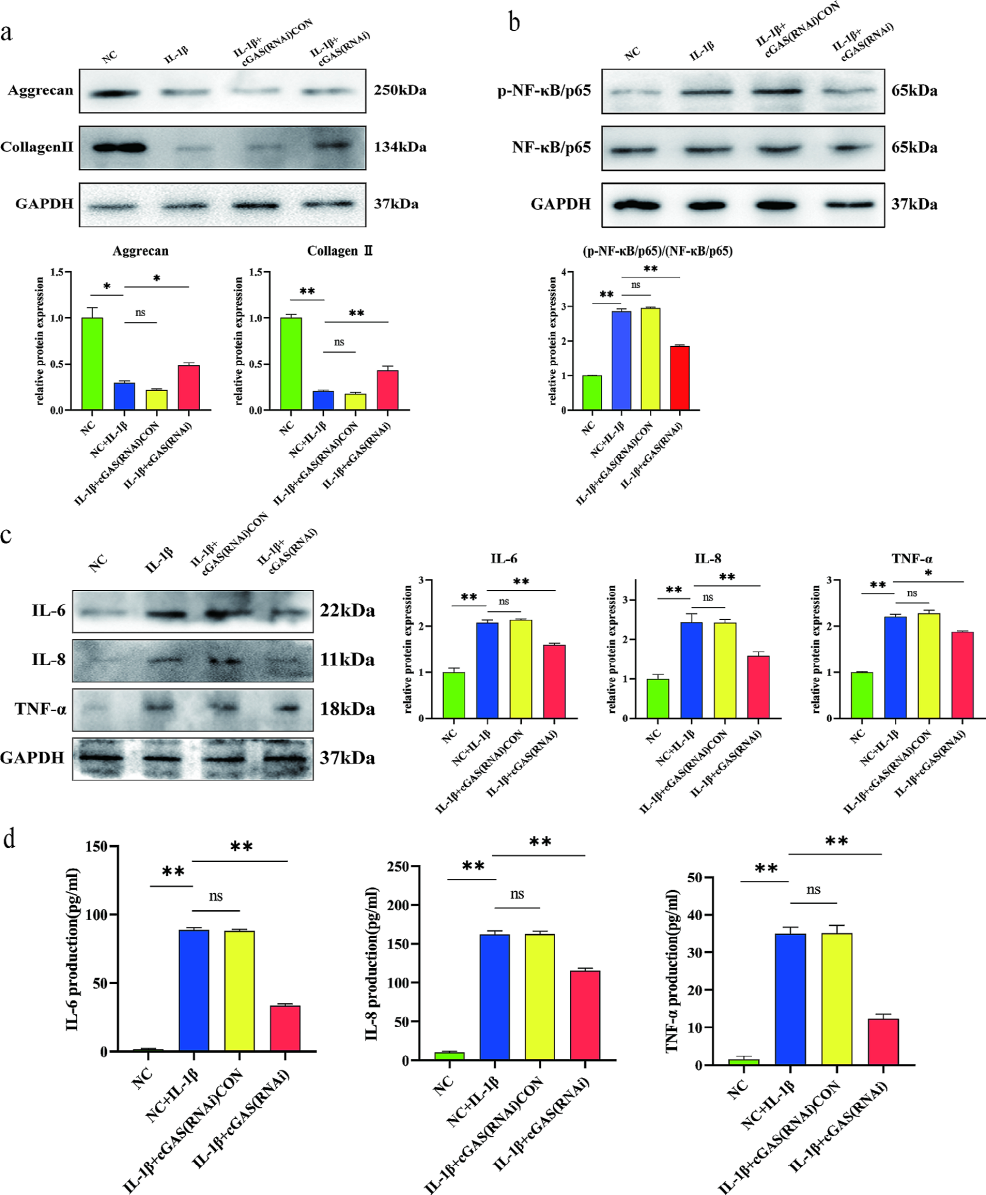
Silencing cGAS alleviated IL-1β induced SASP in NP cells. (**a**) Western blot analysis of aggrecan and collagen II expression in NP cells treated with 10ng/ml IL-1β for 48 h. IL-1β significantly reduced the expression of aggrecan and collagen II in normal or control siRNAs transfected NP cells than in cGAS-silenced NP cells. (**b**) Western blot analysis of NF-κB and p-NF-κB expression. Treatment with 10ng/ml IL-1β for 48 h increased phosphorylated NF-κB in NP cells, which was alleviated by silencing cGAS expression with cGAS-targeting siRNAs. (**c**) Western blot analysis of IL-6, IL-8 and TNF-α expression. Treatment with 10ng/ml IL-1β for 48 h increased IL-6, IL-8, and TNF-α expression in NP cells, which was alleviated by silencing cGAS expression with cGAS-targeting siRNAs. (**d**) ELISA analysis of IL-6, IL-8 and TNF-α expression in NP cells treated with 10ng/ml IL-1β for 48 h. IL-1β significantly increased the secretion of IL-6, IL-8 and TNF-α, which was alleviated by silencing cGAS expression with cGAS-targeting siRNAs. *p<0.05, **p<0.01, ns, not statistically significant

Cultured with IL-1β significantly reduced the expression of aggrecan and collagen but increased IL-6, IL-8, TNF-α, and phosphorylated NF-κB in NP cells (Fig. 4a, b and c). On ELISA analysis, the secretion of IL-6, IL-8 and TNF-α was significantly increased in the NP cells cultured with 10 ng/ml IL-1β for 48 h (Fig. 4d). As compared to the NP cells transfected with non-specific control siRNAs, the NP cells with silenced cGAS reduced the expression and secretion of IL-6, IL-8 and TNF-α (Fig. 4c and d).

## Discussion

Due to permanent cell cycle arrest and SASP-related inflammation of senescent cells, targeting cellular senescence has long been recognized as promising strategy for age-related disease including IVD degeneration [29]. Evidence accumulates that removal of pro-senescent factors or more directly the senescent cells (Senolytics) help to maintain IVD homeostasis [30]. Being a core regulator of cGAS-STING pathways, targeting cGAS to reduce SASP by cGAS-specific small molecule inhibitors has provided new therapeutic approach for senescence-based inflammatory disease. Here we found that cGAS expression was increased in rat degenerated IVD, and inhibiting cGAS in NP cells reduced the expression and secretion of multiple SASP molecules (IL-6, IL-8, TNF-α). Besides, we noticed that cGAS expression was dose-dependently increased by IL-1β in NP cells. Silencing cGAS significantly reduced the expression of senescence pathway molecules (p53, p16) and alleviated cellular senescence induced by IL-1β, suggesting cGAS might also mediate the induction of inflammatory senescence in NP cells. cGAS is interferon (IFN)-stimulated gene and its expression forms positive feedback regulation with IFN signaling and cGAS-STING pathways [31]. The dose-dependent increase of cGAS expression and cellular senescence induced by IL-1β was also evidenced in chondrocytes and the osteoarthritis development in mice [32]. These findings in combination with ours supported a key role of cGAS in potentiating inflammatory senescence and SASP. Targeting cGAS might be a promising anti-senescence strategy for IVD degenerative disease.

In addition to IL-1β, IVD degeneration features an increased expression of multiple pro-inflammatory cytokines including TNF-α, IL-α, IL-6, and IL-17, with NF-κB being a key downstream transcription factor of inflammatory catabolic genes [33]. Here we found that NF-κB was activated in the IL-1β induced NP cell senescence and SASP. Silencing cGAS not only alleviated NP cell senescence but also inactivated NF-κB and reduced the expression of IL-6, IL-8, and TNF-α, suggesting cGAS might be a broad spectrum of target for reducing SASP. In chondrocytes exposed to IL-1β, cGAS and STING were increased and STING promoted extra cellular matrix degradation via NF-κB activation to enhance catabolism while reducing anabolism [34]. Here we showed that slicing cGAS in NP cells reduced NF-κB activation and increased the expression of aggrecan and collagen II, suggesting an anabolic effect of targeting cGAS-STING-NF-κB pathway for restoring IVD matrix. It was reported that activation of TNF-α-NF-κB signals induced Wnt5a expression in NP cells which increased aggrecan and collagen II while suppressing the catabolic TNF-α-NF-κB signaling through a negative-feedback loop. Besides, canonical Wnt/β-catenin signaling was reported to form positive-feedback loop with TNF-α in promoting NP cell senescence and IVD degeneration [35–37]. The crosstalk between NF-κB and Wnt signaling might mediate the anti-catabolic and pro-anabolic effects of silencing cGAS in NP cells.

A recent comparative study of mice constitutively activating STING (STING^+/+^) and STING knockout (STING^−/−^) mice showed that the cGAS-STING pathway was not a critical mediator of senescence onset and consequent degeneration in IVD [38]. As was discussed by the authors the avascular nature of IVD might confer immune privilege and protect it from the STING-induced circulating cytokine storms. In addition, as IVD degeneration is often accelerated by mechanical stress or traumatic injuries, the absence of chemical or mechanical insult in STING^+/+^ and STING^−/−^ mice disc might not fully represent the senescence-based pathophysiology of IVD degeneration. However, in a rat model of vertebral inflammation-induced IVD degeneration, injection of lipopolysaccharide to the vertebrae injures adjacent to endplate significantly activated the cGAS-STING pathway in IVD [39]. In our study cGAS was increased in rat IVD degeneration model induced by annulus puncture, and silencing cGAS protected NP cells from IL-1β induced senescence and SASP. Collectively, targeting cGAS-STING pathway at the early stage of IVD (endplate or annulus fibrosus) injury may halt accelerated disc degeneration, probably by regional alleviation of cellular senescence and SASP-based inflammation.

This study was limited in its ex vivo nature and lack of targeting cGAS for repairing the annulus puncture induced rat IVD degeneration. Future study using STING^+/+^ and STING^−/−^ mice is expected to reveal the role of cGAS-STING pathway in alleviating IVD degeneration accelerated by injury or mechanical stress. Secondly, only inflammatory senescence with IL-1β was investigated. As premature senescence and replicative senescence may encompass different content of DNA damage and cytoplasmic chromatin fragments, it is intriguing to understand the role of cGAS in potentiating cellular senescence and SASP under different pro-senescence conditions. Furthermore, we lack research analysis on the NP tissue of humans. Further validation should be conducted to investigate the differential expression of cGAS in human NP cells.

## Data Availability

No datasets were generated or analysed during the current study.

## Abbreviations

cGAS: Cyclic GMP-AMP synthase
NP: Nucleus pulposus
IVD: Intervertebral disc
SA-β-gal: Senescence-associated beta-galactosidase
SASP: Senescence-associated secretory phenotype
IFN I: Type I interferon
STING: Stimulator of interferon genes
cGAMP: Cyclic GMP -AMP
siRNAs: Small interfering RNAs
